# Optimization of Flavonoid Extraction in* Dendrobium officinale* Leaves and Their Inhibitory Effects on Tyrosinase Activity

**DOI:** 10.1155/2019/7849198

**Published:** 2019-03-13

**Authors:** Haixia Lu, Ke Yang, Lianghui Zhan, Tingting Lu, Xue Chen, Xiamiao Cai, Cong Zhou, He Li, Liuqing Qian, Guiyuan Lv, Suhong Chen

**Affiliations:** ^1^Collaborative Innovation Center of Yangtze River Delta Region Green Pharmaceuticals, No. 18, Chaowang Road, Xiacheng District, Zhejiang University of Technology, Hangzhou, Zhejiang 310014, China; ^2^College of Pharmaceutical Science, No. 548, Binwen Road, Binjiang District, Zhejiang Chinese Medical University, Hangzhou, Zhejiang 310014, China

## Abstract

In order to establish the extraction technology of flavonoids from* Dendrobium officinale* leaves, a method combining Plackett–Burman design (PBD), steepest ascent design, and central composite design was developed to optimize the extraction of flavonoids. In addition, the tyrosinase activity inhibition of flavonoids was further tested in vitro. PBD results showed that ethanol concentration and number of extractions were key factors. Response surface methodology (RSM) indicated that the optimal extraction conditions were 78% ethanol concentration, six extraction times, 2 h, and 1:50 solid-liquid ratio. Under these conditions, the total flavonoid content could reach 35 mg/50 mL. In vitro tyrosinase experiment, the extracted total flavonoids had better inhibitory effect on tyrosinase activity than *β*-arbutin, and its inhibition rate for monophenolase and diphenolase exceeded 100% and 70%, respectively. These results indicate that RSM can effectively improve the extraction of flavonoids from* Dendrobium officinale* leaves and the flavonoids have the prospect of being applied to foods and cosmetics.

## 1. Introduction

Flavonoids, which are a class of compound with a 2-phenylchromanthone structure and C6-C3-C6 as basic carbon scaffold, are usually combined with sugars to form glycosides in plants and a small part in the form of free (glycosides) [1]. Flavonoids have been proven to possess a variety of biological activities, such as antioxidant, anti-inflammatory, and antiapoptosis activities [2, 3]. In the nutraceutical industry, most of the recently discovered natural tyrosinase inhibitors are flavonoids, which are safer and more efficient than certain synthetic tyrosinase inhibitors [4]. Tyrosinase inhibitors can slow down food browning during processing and storage by inhibiting the role of tyrosinase and the growth of a variety of microorganisms [5]. Flavonoids can also be used as whitening agent to achieve whitening efficacy. As the most potential tyrosinase inhibitors, flavonoids have been widely used in foods and cosmetics; hence, finding new and effective tyrosinase inhibitors among natural flavonoids is necessary [6].

*Dendrobium officinale* is a perennial herb in the biological classification of Orchidaceae [7]. Medical history records show that this herb can nourish yin, increase body fluid production, enhance gastric motility, lower blood sugar, and prolong life [8]. As a precious Chinese herb, the stems of* D. officinale* are edible nationally, and the leaves and flowers are also used as food in Zhejiang and Yunnan, respectively. In 2018, the leaves and flowers were included in the “Food Safety Law” for management. With the continuous development of the* D. officinale* industry, the number of people who enjoy eating the leaves and flowers is growing. Our research team has been testing the efficacy of various parts of* D. officinale *for several years, and results show that the chemical composition of each part of* D. officinale* is similar; in particular, the flavonoid content in leaves is higher than that in stems [9]. Flavonoids extracted from the leaves of* D. officinale *have also been found to have antioxidant and antihypertensive effects. In addition, flavonoids may restrain the action of tyrosinase [10], which means that flavonoids can be fully utilized in foods and cosmetics as a tyrosinase inhibitor. However, a universal extraction protocol of flavonoids in the leaves of* D. officinale* does not exist, and its extraction rate is relatively low.

Thus, single-factor experiments were designed to improve the extraction rate of flavonoids in* D. officinale* leaves. On the basis of the results, the extraction process was further optimized by Plackett–Burman design (PBD), steepest ascent design, and response surface methodology. In addition, the inhibition rate of flavonoids on tyrosinase activity was determined in vitro.

## 2. Materials and Methods

### 2.1. Compounds, Materials, and Reagents

*D. officinale* was provided by Zhejiang Senyu Industrial Co., Ltd. (Jinghua, China). Tyrosine, *β*-arbutin, and L-3,4-dihydroxyphenylalanine (L-DOPA) were purchased from Source Leaf Biotechnology Co., Ltd. (Shanghai, China). Ethanol was provided by East China Pharmaceutical Co., Ltd. (Hangzhou, China). A microplate reader was procured from Molecular Devices (Silicon Valley, USA). A UV spectrophotometer was obtained from Shimadzu (Kyoto, Japan). A constant temperature incubator was provided by Shanghai Jinghong Experimental Equipment Co., Ltd. (Shanghai, China), and a rotary evaporator was provided by Henan Yuhua Instrument Co., Ltd. (Henan, China).

### 2.2. Content Determination

Preparation of rutin standard solution was as follows: approximately 10 mg of rutin standard was accurately weighed, placed in a 50 mL volumetric flask, diluted with 70% ethanol to constant volume, and shaken to obtain the mass concentration of 0.2 mg/mL rutin standard solution.

Drawing of the standard curve was as follows: rutin standard solutions of 1.0, 1.5, 2.0, 2.5, 3.0, 3.5, and 4.0 mL were precisely drawn and placed in 10 mL plugged test volumetric flasks. Subsequently, 70% ethanol was added to 5ml; then 0.4 mL of 5% NaNO_2_ solution was added and placed for 6 min. Approximately 0.4 mL of 10% Al (NO_3_)_3_ solution was added and then shaken for 6 min, and 4 mL of 4% NaOH solution was added and placed for 15 min. Finally, the reagent blank was used as reference to measure the absorbance at 510 nm. A standard curve was drawn to obtain a linear regression equation for the rutin standard curve.

Determination of total flavonoids was as follows: the sample was accurately obtained and processed a similar method to the standard. The result was then brought into the standard [11].

### 2.3. Experimental Design

#### 2.3.1. Single-Factor Test

A single-factor experiment was executed to estimate the optimal range of each influencing factor. The effects of the ethanol concentration (35%–85%), number of extractions (1–5), extraction time (0.5–2.5 h), and liquid ratio (1:10–1:60) on the extraction rate of flavonoids were investigated [12, 13]. The amounts of flavonoids were evaluated and analyzed to determine the best parameters for the PBD.

#### 2.3.2. PBD

The effects of four factors on flavonoid extraction were evaluated by the PBD (Table 1). The results of the single-factor experiment confirmed that the selected level of material-to-liquid ratio was 1:40 and 1:60, the extraction time was 1.5 and 2.5 h, the number of extractions was 3 and 5, and the ethanol concentration was 70% and 80%. Twelve groups of experimental conditions were designed by Design Expert V.8.0.6 software [14, 15].

#### 2.3.3. Steepest Ascent Design

The steepest ascent design determined the best areas of these key factors. By analyzing the positive and negative effects on the results of the PBD, the step length and null point of the extraction concentration and number of extractions were 70% and 1.5 and 1 and 1, respectively (Table 2). These values increased the intensity of the experiment, and the area with the best effect was approached [15, 16].

#### 2.3.4. Central Composite Design (CCD)

On the basis of the results of the steepest ascent design, the optimal ethanol concentration was 77.5% and the number of extractions was six [17, 18]. A two-factor CCD, which involved five repetitions of the central point** (**Table 3**), **was devised. In this design, the flavonoid content was the response value, and data analysis was performed for each factor used in Design Expert V.8.0.6 software [19].

#### 2.3.5. Verification and Analysis of Flavonoids in Dendrobium

Three parallel experiments were conducted on the optimal extraction conditions determined by CCD experiments. The resulting values were averaged to obtain the final results.

### 2.4. Determination of Tyrosinase Inhibitory Activity

The obtained extract was concentrated to 0.4 g/mL crude drug, centrifuged for 30 min at 8000 r/min for clarification, and washed off with 50% ethanol by ADS-17 macroporous resin. The extract was concentrated to 2, 3, 4, 5, and 6 mg/mL, and *β*-arbutin was concentrated on the same concentration gradient.

L-Tyrosine and L-DOPA were used as substrates, and four groups of reaction liquids were accurately absorbed into 96-well plates according to the volume presented in Table 4 by using a pipette gun. Tyrosinase was added to each group at 30 *μ*L of 0.25 mg/mL after 10 min of constant temperature in a 37°C constant-temperature incubator. The absorbance of A_0_, A_1_, A_sample_, and A_blank_ at 475 nm after reaction for 10 min, 30 min, 1 h, 2 h, and 4 h was measured by an enzyme labeling instrument. The inhibition rate of tyrosinase activity was calculated using the following formula [20]:

(1) $$\begin{array}{c} Inhibition\;rate\;\left(\;\%\;\right)=\left[\;1-\frac{\left(A_{sample}-A_{blank}\right)}{\left(A_{0}-A_{1}\right)}\;\right]\times 100. \end{array}$$

### 2.5. Statistical Analysis

The results of the PBD, steepest ascent design, and CCD were analyzed by Design Expert.V.8.0.6 software. Other relevant data were analyzed through ANOVA by SPSS 21.0 and considered significant at p < 0.05 and highly significant at p < 0.01.

## 3. Results and Discussion

### 3.1. Single-Factor Experiment of Flavonoid Extraction

**Figure 1:**
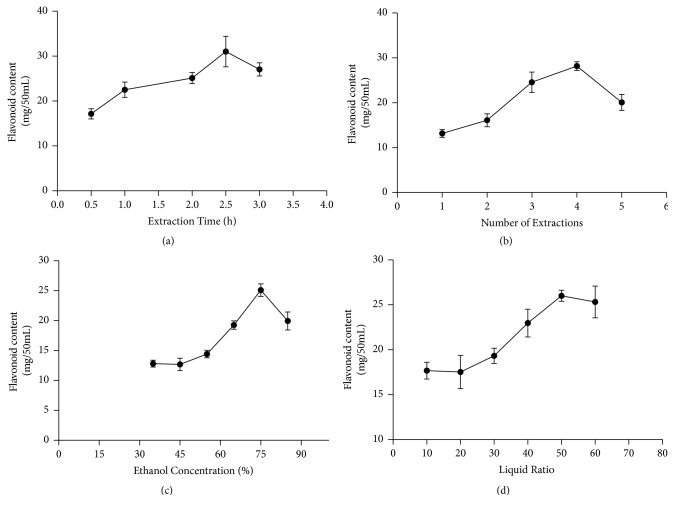
Effect of reaction conditions on the extraction efficiency of flavonoids: (a) extraction time, (b) number of extractions, (c) ethanol concentration, and (d) liquid ratio.

The results of single-factor optimization experiments are shown in Figure 1. The flavonoid content first increased and then decreased with the changes in four various factors. In line with the curve, the extraction times, ethanol concentration, and material-to-liquid ratio exerted evident effects on flavonoid extraction. The optimal conditions were an extraction time of 2.5 h, four extractions, ethanol concentration of 75%, and material-to-liquid ratio of 1:50 [21].

### 3.2. Optimization of Flavonoid Extraction Factors by the PBD

On the basis of the results of single-factor optimization experiments and the influence of extraction conditions, four factors and two levels were used to filter the ethanol concentration, number of extractions, extraction time, and liquid ratio, as shown in Table 1. The ANOVA results are presented in Table 5. Given that a PBD was employed, quadratic polynomial equations with significant terms were obtained as follows:

  Final equation in terms of coded factors:
  Flavonoid = +25.86 + 2.80 × C + 2.15 × D
  Final equation in terms of actual factors:
  Flavonoid = −17.57651 + 2.80253 × Number + 0.42966 × Methanol

In line with the results of the PBD experiment, the regression model obtained by software analysis showed a significant R-squared of 0.8472; thus, the equation was consistent with the actual situation [22]. The two tested factors, namely, the number of extractions and ethanol concentration, significantly (p < 0.01) influenced total flavonoid extraction, and the extraction time and material-to-liquid ratio had no significant effect on the flavonoid content. When the number of extractions was increased from 3 to 5, the flavonoid content significantly increased (p = 0.0003) possibly due to the comprehensive extraction of flavonoids. Changing the ethanol concentration from 70% to 80% may adjust the polarity of the system and alter the solubility of flavonoids [23]. The two factors were assessed as key factors in the steepest ascent design, and the results of single-factor experiment showed that the optimal material-liquid ratio was 1:50 at an extraction time of 2 h.

### 3.3. Optimization of Flavonoid Extraction by the Steepest Ascent Design

The path of steepest ascent was used to approach the optimal region of the two aforementioned factors [24]. On the basis of the experimental results of the previous step, the null point of the number of extractions was 1 (Table 2), the step length was 1, the null point of the ethanol concentration was 70%, and the step size was 1.5. The climbing experiment was set in accordance with the obtained step size and null point, and the results are shown in Table 2.

The results of climbing experiment showed that the flavonoid content gradually increased with the number of extractions and ethanol concentration. When the number of extractions was six and the ethanol concentration was 77.5%, the highest value was reached at 33.81 mg/mL, and the extracted flavonoid content further improved [25, 26].

### 3.4. Optimization of Flavonoid Extraction by the Response Surface Methodology

Through the PBD and steepest ascent design experiment, the significant parameters [27] were selected. Table 3 shows the design group and corresponding results, and Table 6 presents the ANOVA results. The findings suggested that the optimal number of extractions was six, and the ethanol concentration was 78%; the results obtained under the central conditions were similar. Moreover, in accordance with the findings of CCD, the quadratic polynomial equations are expressed as follows:

  Final equation in terms of coded factors:
  Flavonoid = +35.26 +1.16 × A −1.34 × B + 0.36 × A × B − 3.48 × A^2^ − 2.60 × B^2^
  Final equation in terms of actual factors:
  Flavonoid = −9278.13580 + 238.81473 × Methanol + 11.08481 × Number + 0.24249 × Methanol × Number − 1.54512 × Methanol^2^ − 2.60128 × Number^2^

ANOVA was used to evaluate the optimization results. A small p-value indicates a significant influence on the response variables [7]. The model had a correlation p value of 0.0003 < 0.01, which indicated that the optimization model was significant. Furthermore, p values of the number of extractions and ethanol concentration were 0.0207 < 0.05 and 0.0112 < 0.05, respectively, which showed that the two factors exerted significant effects [28]. The final equation in terms of actual factors revealed that the ethanol concentration and number of extractions increased the positive coefficient of the linear effect, whereas A^2^ and B^2^ both had the negative coefficient. The lack of fit was not significant, and the goodness of fit of the model was determined by estimating the variance of the coefficients (R^2^), which was observed as 0.9432; thus, 94.32% of this factor could be interpreted by fitting the model [29]. Moreover, the R-squared value of 0.9432 was close to the adjusted R-squared value of 0.9026, which implied a good statistical model [30].

**Figure 2:**
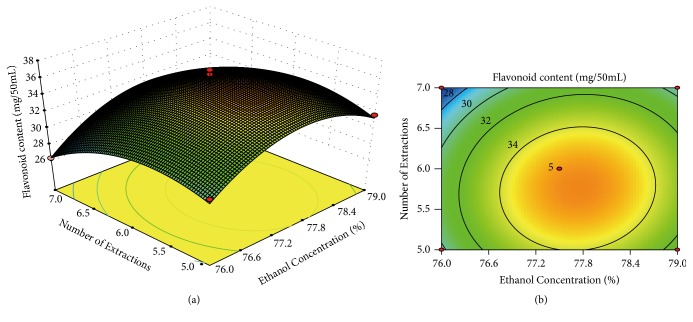
3D response surface plots (a) and 2D contour plots (b).

In this study, 3D curved surface (Figure 2(a)) and 2D contour plots (Figure 2(b)), which explained the relationship between each variable and the experimental level [29] of the corresponding response, were created with extraction times and ethanol concentration as X and Y coordinates, respectively, and flavonoid content as Z coordinates [31]. In the 3D curved surface and 2D contour plots, an interaction was observed between the two selected factors, but the effect was unremarkable [32].

### 3.5. Validation of Optimal Conditions

A confirmatory experiment was performed based on the optimal extraction conditions obtained from the aforementioned experiments. A method was considered credible if the percentage error was less than 10% [7]. The average experimental value for the flavonoid extraction efficiency model was 35.45 ± 0.34 mg/50 mL. A 4.2% difference was observed between the predicted and confirmatory experimental values (Table 7), which confirmed the validity of the optimization model.

In addition, our research group found that the flavonoids in* D. officinale*, which were mostly rutin derivatives, were abundant. As determined via fingerprint analysis, the leaves of* D. officinale *contained eight flavonoids [9], as shown in Table 8.

### 3.6. Determination of Tyrosinase Inhibitory Activity

**Figure 3:**
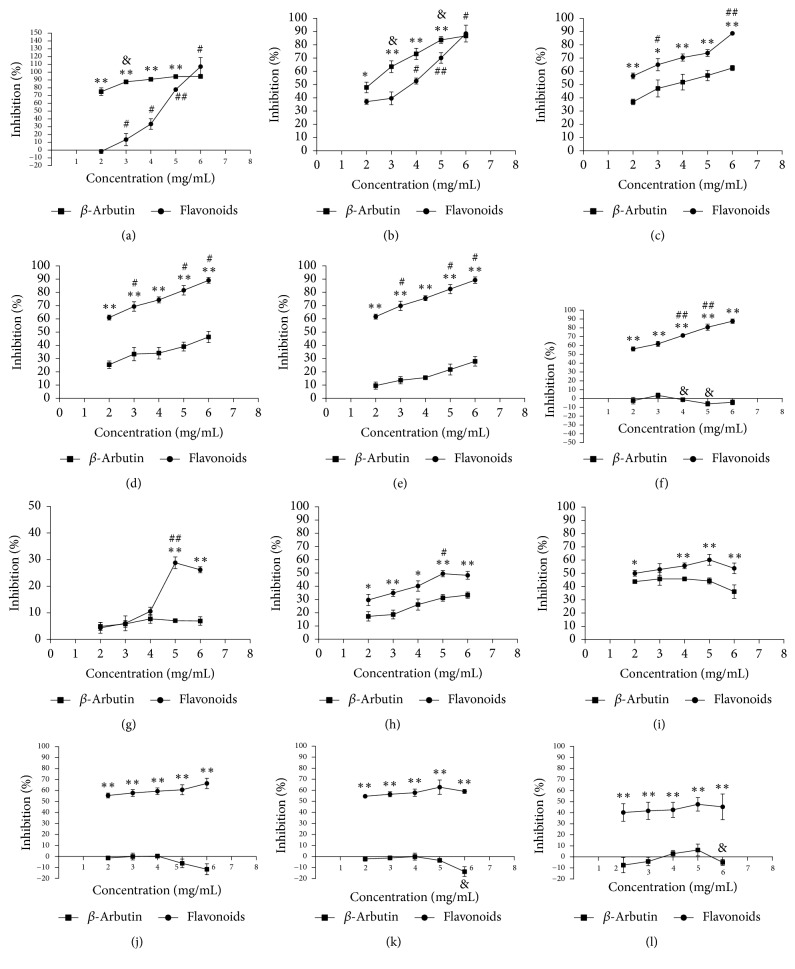
(a)–(f) Monophenolase activity inhibition rates at 10, 30, 60, 90, 120, and 240 min, respectively. (g)–(l) Diphenolase activity inhibition rates at 10, 30, 60, 90, 120, and 240 min, respectively. *∗∗P *< 0.01 versus *β*-arbutin,*∗P *< 0.05 versus *β*-arbutin, ^##^*P *< 0.01, ^#^*P *< 0.05 self-comparison of flavonoids, ^&&^*P *< 0.01,^ &^*P *< 0.05 self-comparison of *β*-arbutin.

L-Tyrosine and L-dopa were used as substrates to detect the inhibitory activity of tyrosinase and *β*-arbutin as positive control [33, 34]. As shown in Figure 3, flavonoid extracts and *β*-arbutin had strong tyrosinase inhibitory activity. Overall, the inhibitory effect of flavonoids was better than that of *β*-arbutin. The trend of the curve revealed that tyrosinase activity was suppressed in a dose-dependent manner. When the concentration of total flavonoids was 6 mg/mL, the inhibitory rate of tyrosine monophenolase was nearly 100%, but the inhibitory rate of tyrosine diphenolase was only 70%. Therefore, compared with the inhibition rate of tyrosine diphenolase (Figures 3(a)–3(f)), the inhibitory effect of flavonoid extract on tyrosine monophenolase (Figures 3(i)–3(l)) was more significant [35].

Six time points were tested to comprehensively understand the inhibition of tyrosine monophenolase activity (Figures 3(a)–3(f)) and diphenolase activity (Figures 3(g)–3(l)) by flavonoid extracts. The inhibitory rate of *β*-arbutin on monophenolase gradually decreased with time, and a slight significant difference was observed among the concentrations. However, the inhibitory rate of flavonoid extracts on monophenolase slowly decreased with the increase in time, and the difference between different concentrations was evident. When the reaction time was 10 min (Figure 3(a)), the inhibition rate of *β*-arbutin was considerably higher than that of flavonoid extracts, except at 6 mg/mL. When the reaction time was 30 min (Figure 3(b)), the inhibition rate of flavonoid extracts was considerably higher than that of *β*-arbutin, and the gap widened with the increase in time; this phenomenon might be related to the strong antioxidant activity of flavonoid extracts for tyrosinase, catalyzing the role of L-tyrosine in the presence of free radicals [36].

Figures 3(g)–3(l) revealed that the effect of flavonoid extracts on diphenolase was more significant compared with that on *β*-arbutin. The best inhibitory effect was observed when the concentration of flavonoid extracts was 5 mg/mL (Figure 3(i)). Similar to the action of monophenolase inhibition, the inhibition rate of diphenolase slightly changed. Therefore, flavonoid extracts exerted better inhibitory effects than *β*-arbutin, and the inhibitory effect on monophenolase was better than that on diphenolase [37].

## 4. Conclusions

This study investigated the extraction of flavonoids by RSM from the leaves of* D. officinale *and analyzed the inhibitory effect of flavonoid extracts on tyrosinase. On the basis of the results of the PBD, steepest ascent design, and CCD, the optimal extraction conditions were determined as follows: ethanol concentration of 78%, six extractions, material-to-liquid ratio of 1:50, extraction time of 2 h, and flavonoid content of 35 mg/50 mL. The extraction of flavonoids from* D. officinale *leaves increased under these conditions.

The results showed that the flavonoid extracts from the leaves of* D. officinale* could effectively inhibit tyrosinase activity, and a dose–effect relationship was observed. At a certain concentration, flavonoid extracts demonstrated good inhibition effect on tyrosine monophenolase and diphenolase. In addition, the inhibition effect and intensity of these extracts were significantly better than those of *β*-arbutin, and their inhibition rate on monophenolase activity was higher than that on diphenolase. Therefore, as a tyrosinase inhibitor, the flavonoid extracts from the leaves of* D. officinale* may become a component in future hypopigmentation foods and cosmetics.

However, this experiment still had some limitations. The composition of flavonoid extracts can be further analyzed in the future, and the types of interaction between flavonoid extracts and tyrosinase can be further researched on cellular and overall levels.

## Figures and Tables

**Table 1:** PBD and results.

Run	Factor 1	Factor 2	Factor 3	Factor 4	Response
	Liquid Ratio (mL/g; X_1_)	Time (h; X_2_)	Number (X_3_)	Ethanol (%; X_4_)	Flavonoid (mg/50mL)
1	1:60	2.5	3	80	24.58
2	1:40	2.5	5	70	24.96
3	1:60	1.5	5	80	32.28
4	1:40	2.5	3	80	26.18
5	1:40	1.5	5	70	24.45
6	1:40	1.5	3	80	22.04
7	1:60	1.5	3	70	22.58
8	1:60	2.5	3	70	20.98
9	1:60	2.5	5	70	27.31
10	1:40	2.5	5	80	30.68
11	1:60	1.5	5	80	32.28
12	1:40	1.5	3	70	21.98

**Table 2:** Path of steepest ascent experimental design and results.

Run	Factor 1	Factor 2	Response
	Number	Ethanol (%)	Flavonoid (mg/50mL)
null point(0)	1	70	
step length(Δ)	1	1.5	
1	1	70	12.94
2	2	71.5	20.26
3	3	73	22.67
4	4	74.5	28.49
5	5	76	30.27
*6*	*6*	*77.5*	*33.81*
7	7	79	31.13

**Table 3:** Central composite design and results.

Run	Factor 1	Factor 2	Response
	Number	Ethanol (%)	Flavonoid (mg/50mL)
1	5	76%	30.22
2	5	79%	31.68
3	7	76%	26.27
4	7	79%	29.18
5	6	75%	26.40
6	6	80%	29.90
7	5	78%	31.40
8	7	78%	28.40
*9*	*6*	*78%*	*36.45 *
*10*	*6*	*78%*	*36.95 *
*11*	*6*	*78%*	*34.13 *
*12*	*6*	*78%*	*33.81 *
*13*	*6*	*78%*	*34.95 *

**Table 4:** Loading table.

Reagent /Number	A_0_	A_1_	A_Sample_	A_blank_
L-tyrosine /L-dopa	100	0	100	0
Tyrosinase	30	30	30	30
Inhibitor	0	0	10	10
Buffer	70	170	60	160

**Table 5:** ANOVA and R-squared for selected factorial model.

Source	Sum of squares		Mean	F	p-value	
		df	Square	Value	Prob>F	
Model	149.63	2	74.82	24.96	0.0002	significant
C-number	94.25	1	94.25	31.44	0.0003	
D-Methanol	55.38	1	55.38	18.48	0.002	
Residual	26.98	9	3.00			
Cor Total	176.61	11				
Std. Dev.	1.73					
Mean	25.86					
C.V.%	6.70					
PRESS	47.96					
R-Squared	0.8472					
Adj R-Squared	0.8133					
Pred R-Squared	0.7284					
Adeq Precisior	11.438					

**Table 6:** ANOVA and R-squared for the selected factorial model.

Source	Sum of squares		Mean	F	p-value	
		df	Square	Value	Prob>F	
Model	142.41	5	28.48	23.24	0.0003	Significant
A- Methanol	10.85	1	10.85	8.85	0.0207	
B- number	14.31	1	14.31	11.68	0.0112	
AB	0.53	1	0.53	0.43	0.5321	
A^2^	84.08	1	84.08	68.59	<0.0001	
B^2^	47.07	1	47.07	38.40	0.0004	
Residual	8.58	7	1.23			
Lack of Fit	0.84	3	0.28	0.15	0.9275	Not Significant
Pure Error	7.74	4	1.93			
Cor Total	150.99	12				
Std. Dev.	1.11					
Mean	31.52					
C.V.%	3.51					
PRESS	18.09					
R-Squared	0.9432					
Adj R-Squared	0.9026					a
Pred R-Squared	0.8802					
Adeq Precisior	11.890					

**Table 7:** Verification of the experimental results.

Category	Run	Yield (mg/50mL)		STDEV (%)
		Predictive	Experimental	
Flavonoids	1	35.51	35.41	4.2%
	2	35.51	35.13	
	3	35.51	35.81	

**Table 8:** Flavonoids in *D. officinale.*

Herb	Flavonoids
*D. officinale*	Apigenin-6, 8- two -C-*β*-D- glucoside
	Apigenin-6-C-*β*-D-xylose-8-C-*β*-D-glucopyranoside
	Isophora
	Schaftoside
	Apigenin-6-C-*β*-D- glucoside -8-C-*β*-D-glucoside
	Apigenin-6-C-*β*-D-xylose-8-C-*α*-L-arabinoside
	Apigenin-6,8-di-C-*α*-L-pyranosin
	Apigenin-6-C-*α*-L-arabinose-8-C-*β*-D-xyloside

## Data Availability

The data used to support the findings of this study are available from the corresponding author upon request.
